# Development of a Sensitive and Reliable UHPLC-MS/MS Method for the Determination of Multiple Urinary Biomarkers of Mycotoxin Exposure

**DOI:** 10.3390/toxins12030193

**Published:** 2020-03-18

**Authors:** Zhezhe Liu, Xiaoxue Zhao, Libiao Wu, Shuang Zhou, Zhiyong Gong, Yunfeng Zhao, Yongning Wu

**Affiliations:** 1 School of Food Science and Engineering, Wuhan Polytechnic University, Wuhan 430023, China; 2 NHC Key Laboratory of Food Safety Risk Assessment, Food Safety Research Unit (2019RU014) of Chinese Academy of Medical Science, China National Center for Food Safety Risk Assessment, Beijing 100021, China

**Keywords:** human biomonitoring, emerging mycotoxins, metabolites, biomarkers, urine, UHPLC-MS/MS

## Abstract

A variety of mycotoxins from different sources frequently contaminate farm products, presenting a potential toxicological concern for animals and human. Mycotoxin exposure has been the focus of attention for governments around the world. To date, biomarkers are used to monitor mycotoxin exposure and promote new understanding of their role in chronic diseases. The goal of this research was to develop and validate a sensitive UHPLC-MS/MS method using isotopically-labeled internal standards suitable for accurate determination of 18 mycotoxin biomarkers, including fumonisins, ochratoxins, *Alternaria* and emerging *Fusarium* mycotoxins (fumonisin B_1_, B_2_, and B_3_, hydrolyzed fumonisin B_1_ and B_2_, ochratoxin A, B, and alpha, alternariol, alternariol monomethyl ether, altenuene, tentoxin, tenuazonic acid, beauvericin, enniatin A, A_1_, B, and B_1_) in human urine. After enzymatic digestion with β-glucuronidase, human urine samples were cleaned up using HLB solid phase extraction cartridges prior to instrument analysis. The multi-mycotoxin and analyte-specific method was validated in-house, providing satisfactory results. The method provided good linearity in the tested concentration range (from LOQ up to 25–500 ng/mL for different analytes), with R^2^ from 0.997 to 0.999. The limits of quantitation varied from 0.0002 to 0.5 ng/mL for all analytes in urine. The recoveries for spiked samples were between 74.0% and 133%, with intra-day precision of 0.5%–8.7% and inter-day precision of 2.4%–13.4%. This method was applied to 60 urine samples collected from healthy volunteers in Beijing, and 10 biomarkers were found. At least one biomarker was found in all but one of the samples. The high sensitivity and accuracy of this method make it practical for human biomonitoring and mycotoxin exposure assessment.

## 1. Introduction

Mycotoxins are toxic secondary metabolites produced under favorable conditions by different species of fungi that can grow in a wide variety of cereals and foods from production to storage [1,2]. Their occurrence and concentrations vary considerably among foods due to factors such as crop susceptibility, climate change, storage and transportation conditions, as well as sanitary standards [3,4]. Mycotoxins are hepatotoxic, nephrotoxic, teratogenic, carcinogenic, cytotoxic, immunosuppressive, inflammatory, neurotoxic, and estrogenic, posing diverse health hazards to humans and animals [5,6,7,8]. In particular, fumonisins (FBs) and ochratoxin A (OTA) have been classified as possible human carcinogens (Group 2B). These major mycotoxins have been strictly regulated by the Joint FAO/WHO Expert Committee on Food Additives (JECFA), and many countries have set maximum levels (MLs) in food and feed [9,10].

With climatic change, some emerging mycotoxins, *Alternaria* toxins, and *Fusarium* mycotoxins (enniatins and beauvericin) have been found in foods. *Alternaria* toxins, including alternariol (AOH), alternariol monomethyl ether (AME), altenuene (ALT), tentoxin (TEN), and tenuazonic acid (TeA), are produced by *Alternaria* species and have strong evidence of acute and chronic toxicity [11]. The most well-known enniatins (ENNs) reported as natural contaminants are ENNA, ENNA1, ENNB, and ENNB1, which have shown cytotoxic and apoptotic activities [12]. Studies published to date have rarely paid close attention to *Alternaria* and emerging *Fusarium* mycotoxins, despite several scientific reports published by the European Food Safety Authority (EFSA) [11,12]. Regulations or MLs for these emerging toxins in food and feed have not been established due to the absence of comprehensive occurrence data and toxicological characterization. Therefore, it is imperative to collect their occurrence and exposure data worldwide.

Mycotoxin exposure assessment is traditionally based on calculations combining mycotoxin contamination in food data with population food consumption data [13]. Taking into account the heterogeneous distribution of mycotoxins in food, the modified forms that cannot be determined, and the sources other than dietary intake (i.e., inhalation and occupational exposure), human biomonitoring (HBM) using biomarkers of exposure in biological fluids has been accepted as a suitable alternative to assess the aggregated exposure to mycotoxins from different origins for a more accurate and comprehensive assessment at the national, regional, or even individual levels [14,15,16]. Exposure biomarkers of mycotoxins include the parent compounds themselves, the metabolites formed in vivo or products that interact with macromolecules, such as DNA or proteins [17]. Urine, faeces, blood, breast milk, or hair may be selected as biological samples for biomarker analysis. Urine is the preferred sample matrix, as it is easy to collect, readily available, and easy to handle. Urinary biomarkers usually provide information about recent intake, especially for mycotoxins possessing high excretion rates through the kidneys [18].

Non-metabolized fumonisins B_1_ (FB_1_) and ochratoxin A (OTA) are important urinary biomarkers [19]. Moreover, ochratoxin alpha (OT-alpha) is also a well-known metabolite of OTA generated by segmentation of the peptide bond of OTA in vitro [20]. Muñoz et al. suggested that OT-alpha could be a sensitive biomarker [21]. Both OTA and OT-alpha are excreted mainly by urine in humans [20]. Fumonisin B_1_ (FB_1_), fumonisin B_2_ (FB_2_), and fumonisin B_3_ (FB_3_) are naturally present in corn or corn-based products [22]. Fumonisins are highly stable in vivo and are mainly excreted via the fecal route, with less than 3% recovered in urine [23,24,25]. A portion of fumonisins can also be degraded to hydrolyzed fumonisins in human microsomes [26]. Hydrolyzed fumonisin B_1_ (HFB_1_) has been found in the gut of vervet monkeys and can be used as an additional biomarker [27,28].

The urinary excretion of TeA was confirmed to be close to 100%. Some studies concluded that efficient urinary excretion of AOH and AME were 9% and over 2.6% after 24 h, respectively. AOH has been used as a biomarker of exposure in human urine samples [3]. Some cell and animal experiments identified the metabolites of AOH and AME to be AOH-3GlcA, AOH-7GlcA, AOH-9GlcA, AME-3GlcA, and AME-7GlcA [11]. ENNs and beauvericin (BEA) are bioactive compounds. For ENNs, since the information about their metabolism is still scarce, determination of parent compounds is the current evaluation method [7].

According to the description above, mycotoxin biomarkers are excreted in urine in the form of free and conjugated forms. Generally, conjugated forms cannot be quantified directly because of the lack of standard materials [29]. Glucuronidation is one of the main phase II metabolic pathways in the human body. Mycotoxin-glucuronides can be formed in liver and excreted in urine [30]. Hence, in this study, urine samples were digested with β-glucuronidase to break down the conjugated forms and obtain more accurate exposure results.

Among the published methods for mycotoxin determination in biological samples, including HPLC [20,31,32], GC-MS/MS [33], LC-MS [34,35], LC-MS/MS [4,18,36,37], and LC-HRMS [38], LC-MS/MS provides remarkable selectivity, accuracy and sensitivity. Most of the methods were applied to detect common regulated mycotoxins [4,5,8,19,32,37,39,40,41,42], employing various sample preparation strategies such as the “dilute and shoot” approach [4,5,37,41], QuEChERS [15], liquid–liquid extraction (LLE) [20], immunoaffinity (IAC) columns [41,42], solid phase extraction (SPE) [18], and various combinations of these techniques [13]. However, only a few methods targeted for *Alternaria* and emerging *Fusarium* mycotoxins, including ENNB [38,43], AOH [3], AOH and AME [15], TeA [44], ENNs, and BEA [7], for human biomonitoring.

To expand the research scope, acquire more extensive occurrence data about multiple mycotoxins and characterize their potential risks, we developed a method for simultaneous quantitation of 18 mycotoxin biomarkers, including common toxins and emerging toxins in urine. The method provided satisfactory recovery and precision for human biomonitoring. Isotope internal standards were used to ensure accuracy and effectively compensate for changes in matrix effects, extraction, and even other unperceived potential interference problems. Furthermore, this method covered a major category of mycotoxin metabolites, the glucuronide conjugates, by using β-glucuronidase to release them into free forms.

## 2. Results and Discussion

### 2.1. Sample Clean-Up

When simultaneously determining multiple target analytes, sample pretreatment steps are critical to overcome matrix interference, and this process can improve method recovery and sensitivity in UHPLC-MS/MS detection at the same time, especially for complex samples such as urine and serum. Urine contains many impurities that vary in composition and concentration. Several cartridges, such as Mycosep 226, Multisep^®^ 211 Fum, and Oasis HLB, have been successfully applied to mycotoxin analyses in food, feed, serum, and urine. Mycosep and Multisep cartridges are multi-functional columns containing a combination of adsorbents that was specifically designed for mycotoxin analysis. These cartridges can retain interfering substances from complex samples and allow analytes of interest to pass through.

**Figure 1:**
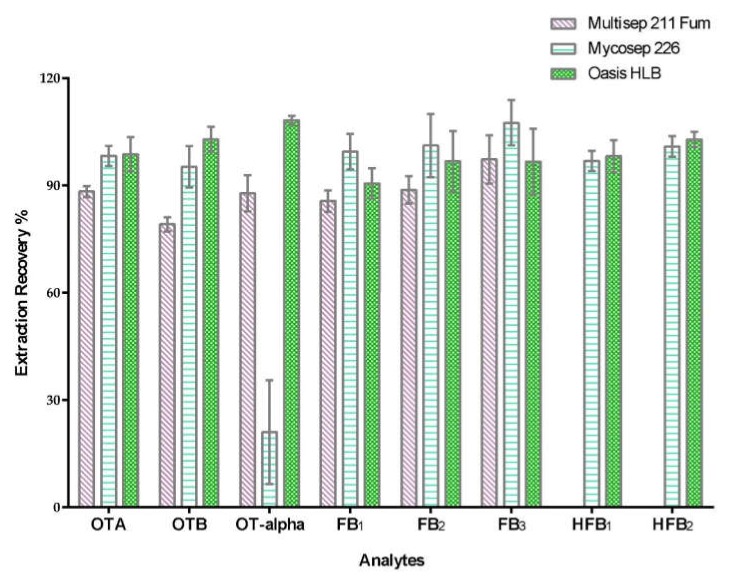
Extraction recovery using Multisep 211 Fum, Mycosep 226 and Oasis HLB cartridge for 8 mycotoxins (OTA, OTB, OT-alpha, FB_1_, FB_2_, FB_3_, HFB_1_, and HFB_2_) spiked at 10 ng/mL.

Hence, these cartridges were evaluated in this work. A working solution of standard mixture was used to compare the recoveries of the target analytes. Figure 1 shows the percent recovery obtained from different cartridges for 8 analytes (ochratoxins and fumonisins). Other 10 analytes (*Alternaria* toxins, ENNs and BEA) were optimized in our laboratory, and the results were published previously [45]. We compared the performance of Mycosep 226, Oasis C18 cartridges, and Oasis HLB and their ability to enrich the 10 compounds. Better recoveries were obtained when using the Oasis HLB column.

**Figure 2:**
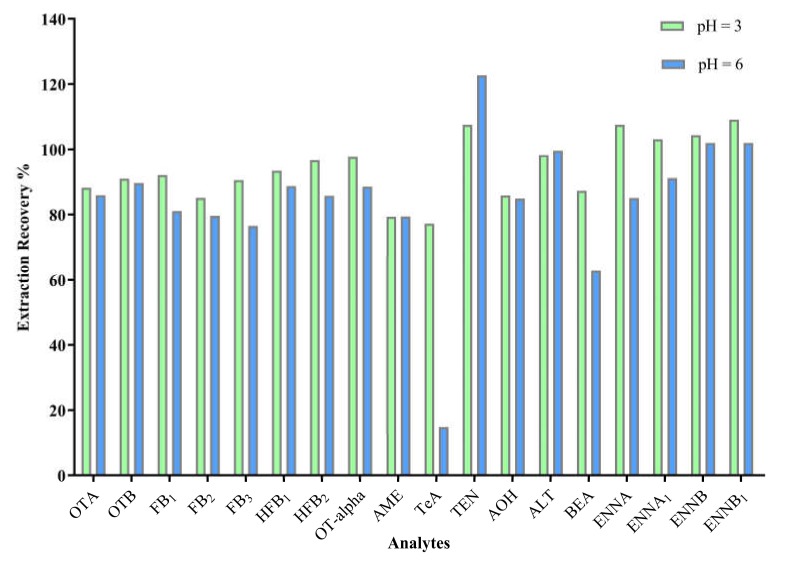
Extraction recovery using Oasis HLB cartridge at different pH values for 18 target mycotoxins spiked at 10 ng/mL.

Under normal dietary conditions, the pH of human urine is 4.6–8.0. According to the instructions of the HLB column, samples were acidified with formic acid before loading. Then, the pH value of loading sample was optimized to obtain maximum and stable retention of 18 analytes on the HLB cartridge. As shown in Figure 2, pH 3 gave a satisfactory result. At pH 6, TeA is in an ionic state and cannot be well retained by the HLB cartridge.

**Figure 3:**
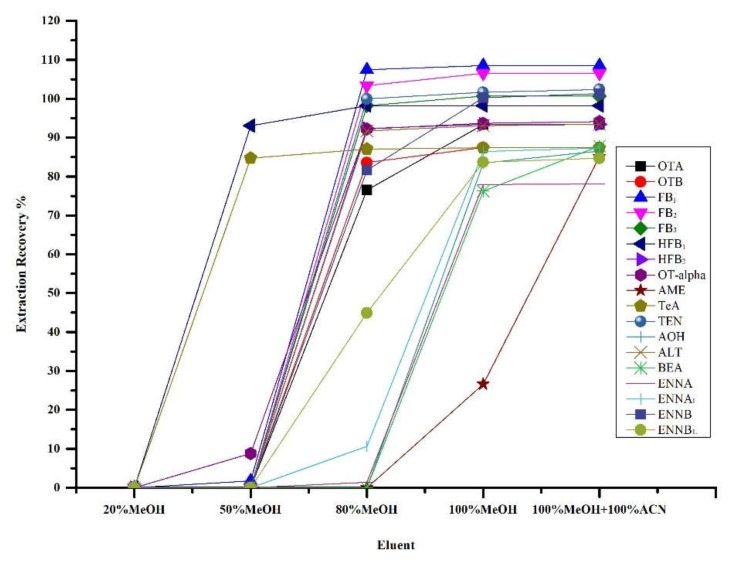
Recovery using different eluent for 18 mycotoxins spiked at 10 ng/mL.

Urine contains multiple endogenous water-soluble components, including mineral salts, hormones, vitamins, amino acids, urea, creatinine, and other metabolites, which may cause complex interferences and column clogging. As shown in Figure 3, different eluents (20% MeOH, 50% MeOH, 80% MeOH, 100% MeOH and equal proportion of MeOH and ACN, v/v) were tested. The solution containing 20% MeOH was selected as the wash solution to reduce impurities and increase analyte recovery. Five milliliters of MeOH and then 5 mL of ACN were used as the eluent, to obtain the highest recovery for most target compounds. The eluate was dried under nitrogen gas and reconstituted in 0.2% FAc/ACN (50/50, v/v).

### 2.2. Enzyme Hydrolysis

After ingestion, a portion of mycotoxin can be metabolized to glucuronides and excreted into urine. To obtain the total (free + glucuronides) level of urinary biomarkers, an enzyme hydrolysis using β-glucuronidase was performed to break down the conjugated forms and obtain more accurate exposure results. The most highly contaminated urine sample (containing 54.1 ng/mL of total TeA) found in our study was selected and used to evaluate the completeness of enzyme digestion. To 1 mL of the urine sample, 1 mL of enzyme solution containing different amounts of β-glucuronidase (200, 500, 1000, 2000, and 5000 units) was added and incubated at 37 °C overnight. The concentration of TeA was quantified, which increased along with the amount of β-glucuronidase and reached a platform at 1000 units. It indicated that 1000 units β-glucuronidase per mL urine was sufficient for the maximum release of TeA. Finally, 2000 units/mL urine was chosen in the study.

### 2.3. Optimization of MS-MS Parameters

For each analyte, more than three different multiple reaction monitoring (MRM) transitions were optimized via the injection of individual standard solutions. Key parameters that influence sensitivity for different MRM transitions were manually optimized, including declustering potential (DP) and collision energy (CE). To ensure sufficient sample ionization, a lower DP was selected to increase the abundance of the parent ion, and the total sensitivity increased correspondingly. For AOH, TeA, AME, and OT-alpha, the signal intensity was relatively higher in negative ion mode than in positive ion mode. Selection of product ions and optimization of CE were performed by individual infusions of each analyte standard. During the infusion, CE was tuned from 10–60 eV to produce the most intensive and stable product ion signal in real-time product-ion scan spectrum. Finally, two transitions with the greatest sensitivity and minimum impurity interference were selected for each compound. Each MS/MS transition and its corresponding optimal DP and CE are shown in Table 1.

The ion source parameters, including ionization mode, curtain gas (CUR), collision gas (CAD), ion spray voltage (IS), temperature (TEM), ion source gas 1 (GS1), and ion source gas 2 (GS2), were manually optimized for better sensitivity (S/N) for most compounds, and the results are summarized in Table 2.

### 2.4. Optimization of Chromatographic Separation

**Figure 4:**
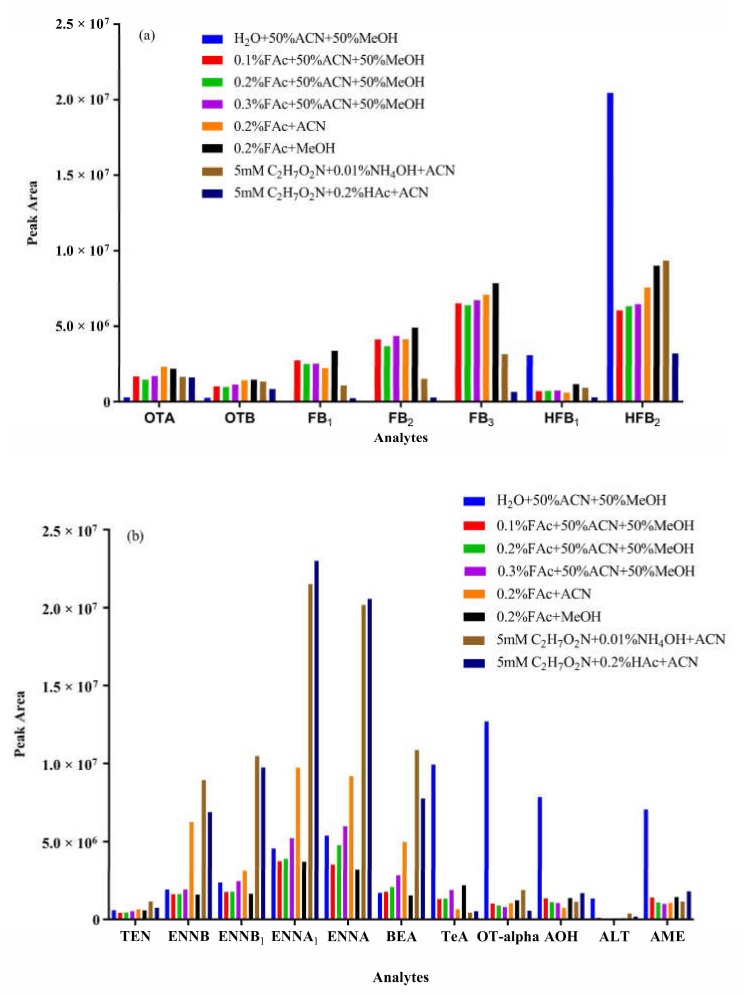
Evaluation of the effects of additives in the mobile phase on the peak areas for (**a**) 7 analytes (OTA, OTB, FB_1_, FB_2_, FB_3_, HFB_1_, and HFB_2_) and (**b**) 11 analytes (OT-alpha, ATs, ENNs, and BEA). Abbreviations: FAc, formic acid; HAc, acetic acid; C_2_H_7_O_2_N, ammonium acetate; NH_4_OH, ammonia water solution; ACN, acetonitrile; MeOH, methanol.

**Figure 5:**
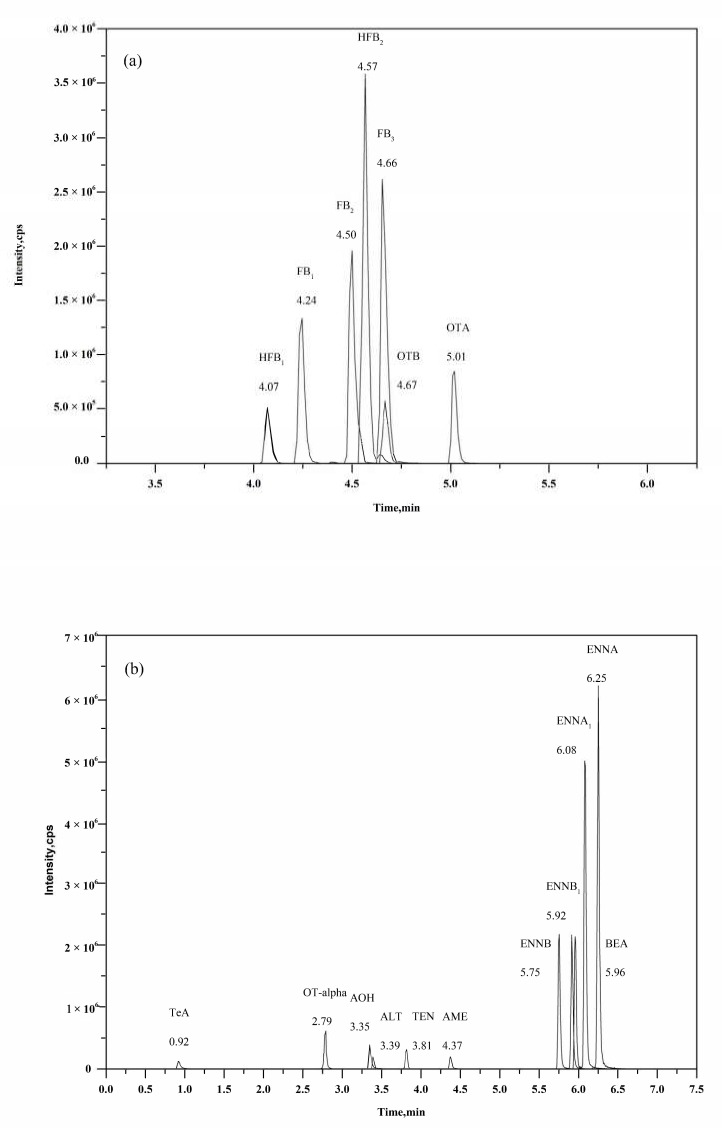
MRM-chromatograms of (**a**) a standard mixture of 7 mycotoxins (2 ng/mL OTA, 10 ng/mL OTB, 10 ng/mL FB_1_, 10 ng/mL FB_2_, 10 ng/mL FB_3_, 5 ng/mL HFB_1_, and 10 ng/mL HFB_2_) and (**b**) a standard mixture of 11 mycotoxins (5 ng/mL OT-alpha, 10 ng/mL AOH, 20 ng/mL TeA, 20 ng/mL ALT, 2 ng/mL AME, 2 ng/mL TEN, 1 ng/mL ENNB, 1 ng/mL ENNB_1_, 2 ng/mL ENNA, 2 ng/mL ENNA_1_, and 2 ng/mL BEA).

Chromatographic separation is the next equally crucial part in a reliable UHPLC-MS/MS method. Chromatographic performance was optimized, including the column, mobile phase and its additives, flow rate, elution gradient, and proper column temperature. The CORTECS C18 UPLC column was selected due to its 1.6 μm core-shell packing particles that provide a more effective separation of the analytes in a shorter run-time, compared to a porous column (BEH C18 UPLC column). After the mobile phase was optimized (Figure 4), the 18 analytes were divided into two groups for instrument analysis. For the first group of compounds (OTA, OTB, FB_1_, FB_2_, FB_3_, HFB_1_, and HFB_2_), acetonitrile gave a lower background signal and stronger elution ability than methanol. A ratio of 1:1 methanol (MeOH) to acetonitrile (ACN) was chosen as solvent B, for optimal separation efficiency and sensitivity. As shown in Figure 4a, an acidic mobile phase is necessary for most ochratoxins and fumonisins, since H^+^ can improve their ionization efficiency. More importantly, satisfactory chromatographic separation of fumonisins can easily be achieved in acid conditions. Even though, different proportions of aqueous formic acid (FAc) had little effect on the sensitivity. HFB_1_ and HFB_2_ had larger peak areas in neutral condition. However, they displayed tailing peaks in neutral and alkaline conditions, while gave sharp peaks in acidic mobile phase. As a consequence, HFB1 and HFB2 were classified into the first group using 0.2% FAc and MeOH:ACN (1:1, v:v) as an optimal mobile phase. Seven analytes achieved satisfactory separation, as displayed in Figure 5a. For the second group (OT-alpha, *Alternaria* toxins, ENNs, and BEA), acetonitrile was used as solvent B. Ammonium acetate, ammonium formate, and ammonium hydroxide at different concentrations were evaluated as additives in the aqueous phase. TeA, OT-alpha, ALT, AOH, and AME had the largest peak areas in water with no additives, but obvious peak broadening and tailing were observed. As a result, the aqueous phase contained 5 mmol/L ammonium acetate and 0.01% ammonia was used as the optimal solvent A for the second group, after comprehensive consideration of peak shape and mass signal intensity. The results are shown in Figure 4b. The representative chromatogram of the second group is presented in Figure 5b.

### 2.5. Validation Experiments

The linearity, method recovery, intra-day and inter-day precision, limit of detection (LOD), and limit of quantification (LOQ) were evaluated for the established method (involving enzyme hydrolysis, SPE purification, and UHPLC-MS/MS analysis), following the guidelines of Commission Decision 200/657/EC [46], EMEA [47], and FDA [48].

Calibration curves prepared in pure solvent (ACN/0.2%Fac, 50/50) were linear from LOQ up to 25 ng/mL for ENNB and ENNB_1_, 50 ng/mL for ENNA, ENNA_1_, TEN, AME, and OTA, 125 ng/mL for HFB_1_ and OT-alpha, 250 ng/mL for FB_1_, FB_2_, FB_3_, HFB_2_, and AOH, and 500 ng/mL for the remaining mycotoxins with 1/x^2^ weighting (Table 3). Linearity was assessed on three consecutive days, with the average correlation coefficients in range of 0.997 to 0.999, which demonstrated good linear responses for all analytes.

Method recovery (R_M_) and precision values were determined from spiked urine samples at three (low, medium, and high) levels with internal standards correction, as described in the Section 4. Nearly all the analytes displayed acceptable recoveries with internal standard correction, ranging from 74.0% to 133%. The intra-day precision and inter-day precision were 0.5%–8.7% and 2.4%–13.4%, respectively. The recovery and precision data for all concentrations are shown in Table 4.

LOD and LOQ of each analyte in urine was evaluated by spiking urine matrix at low concentration levels that generated signal-to-noise ratios (S/N) of 3 and 10, respectively. The LOQ ranged from ppt levels (0.2 pg/mL for ENNB) to ppb levels (0.5 ng/mL for TeA). The other detailed information has been listed in Table 3. This method showed improved LOD and LOQ compared to published methods for the same compounds in urine, which is summarized in Table 5.

Matrix effects (SSE), extraction recovery (R_E_), and apparent recovery (R_A_) were also determined to evaluate the sample preparation step (including enzyme hydrolysis) and instrumental detection step separately. It was performed using three sets of calibration curves without internal standard correction as described in the Section 4. As shown in Table 3, due to the complexity of the matrix, the SSE ranged from 12.2% to 119.6%. There was severe signal suppression for OT-alpha and AOH. R_E_ values were satisfactory for all the analytes in a range of 71.6%~111%. The results demonstrated an effective analyte extraction, while showing the necessity of internal standard compensation. Based on the results, internal standards with similar R_A_ were selected as reference internal standards for compounds lacking commercial internal standards. ^13^C-FB_2_ was used as the reference internal standard for HFB_1_, HFB_2_, and OTB quantification. AME-d_3_ was selected for ENNs and BEA quantification. ^13^C-FB_1_ and AOH-d_2_ were employed to more accurately quantitate ALT and OT-alpha after evaluation, respectively. The R_E_ was 71.6%–110.8%, indicating great extraction efficiency of sample preparation.

### 2.6. Application of the Method

**Figure 6:**
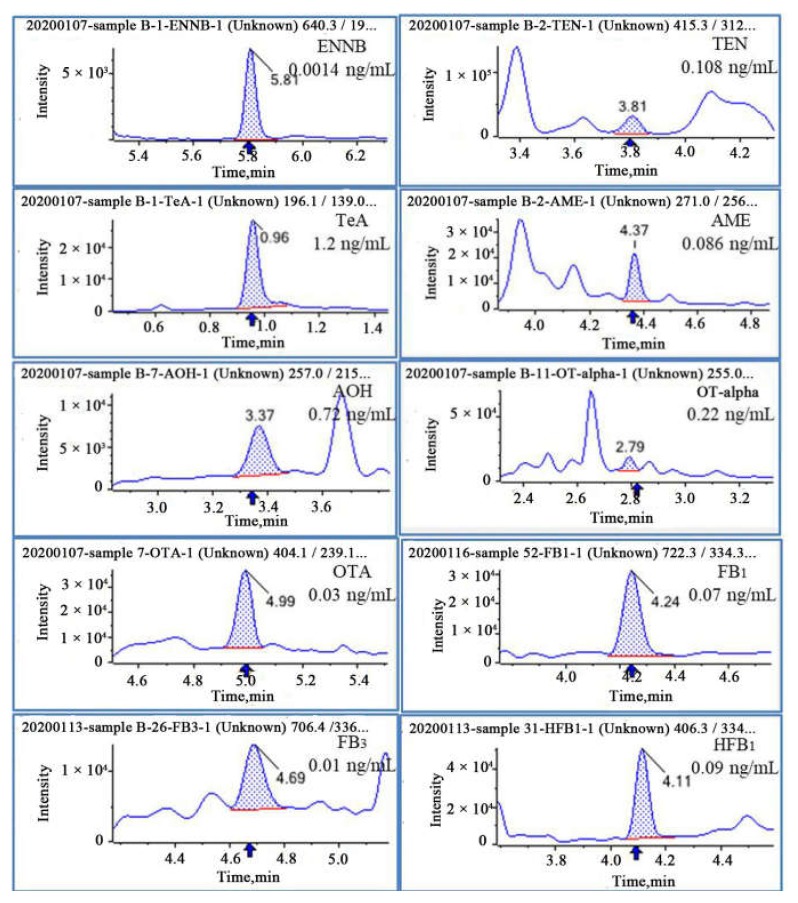
Representative MRM-chromatograms of urine samples with natural contamination of mycotoxins. ENNB, TEN, TeA, AME, AOH, FB_1_, FB_3_, HFB_1_, OT-alpha, and OTA were found.

Sixty samples of human urine were analyzed in duplicate using the optimized method. QC samples were included in each batch of analysis, and their measured values should be within ±15% of the theoretical values. After β-glucuronidase digestion, the results revealed that 98.3% of the analyzed samples contained at least one mycotoxin, and 35 urine samples (58.3%) contained four or more mycotoxins. Overall, *Alternaria* mycotoxins were highly detected followed by ochratoxins. The most frequently detected was TeA (86.7%), with concentrations of 4.4 ± 8.7 ng/mL. AME had the second highest detection rate at 83.8% of samples, with concentrations ranging from <LOD to 0.167 ng/mL. Similar detection rates of TEN (38.3%) and AOH (28.3%) were observed. OTA and OT-alpha were found in about one-third of the samples with the mean concentrations of 0.02 ± 0.02 and 0.22 ± 0.42 ng/mL, respectively. ENNB (40.0%) were the most frequently detected *Fusarium* toxins. FB_1_ (3.3%), FB_3_ (1.7%), and HFB_1_ (1.7%) were rarely detected. The remaining ENNs, OTB, FB_2_, HFB_2_, and ALT were not detected in any of the analyzed samples. The relative intensities of two product ions and the retention time (RT) were used to identify each compound, according to the Commission Decision 2002/657/EC. The results of all samples are summarized in Table 6, demonstrating sufficient sensitivity and applicability of this method for human biomonitoring. Several representative MRM-chromatograms of naturally contaminated samples are displayed in Figure 6.

## 3. Conclusions

This study reports on the development of an accurate and sensitive UHPLC-MS/MS method for the determination of 18 mycotoxins in human urine. The recoveries of target analytes ranged from 74.0% to 133%, with inter-day RSD being less than 13.4%. These values are within the acceptable range. After optimization, the LOQs were in the range of 0.0002 to 0.5 ng/mL, exhibiting good sensitivity. The method was optimized and successfully applied to human urine analysis. Ten of the 18 biomarkers were detected, and at least one biomarker was found in all but one of the samples. In summation, the optimized analytical strategy represents a reliable tool for mycotoxin exposure assessment and contributes to more relevant studies. In the analysis of 60 urine samples, the high occurrence and concentration levels of TeA, AME, and ochratoxins are worthy of attention in future risk assessment studies.

## 4. Materials and Methods

### 4.1. Reagents and Chemicals

The following mycotoxin and isotopically-labeled mycotoxin standard solutions were purchased from Romer labs (Tulln, Lower Austria, Austria): OTA, OTB, OT-alpha, FB_1_, FB_2_, FB_3_, HFB_1_, AOH, AME, ALT, TEN, TeA, BEA, ENNA, ENNA_1_, ENNB, ENNB_1_, ^13^C_20_-OTA, ^13^C_34_-FB_1_, ^13^C_34_-FB_2_, and ^13^C_34_-FB_3_. Solid powder of the HFB_2_ standard, as well as TEN-d_3_, AOH-d_2_, TeA-d_13_, and AME-d_3_, were obtained from Toronto Research Chemicals (TRC, Toronto, ONT, Canada). Solid standard substances were dissolved in pure acetonitrile (ACN) or ACN/water (50/50). Individual standard solutions were kept at −30 °C According to the different sensitivities of each analyte, a multi-standard stock solution was prepared in ACN/water (50/50) containing OTA (0.5 μg/mL), HFB_1_ (1.25 μg/mL), OTB (2.5 μg/mL), FB_1_ (2.5 μg/mL), FB_2_ (2.5 μg/mL), FB_3_ (2.5 μg/mL), and HFB_2_ (2.5 μg/mL). A second multi-standard stock solution was prepared in pure ACN containing ENNB (0.25 μg/mL), ENNB_1_ (0.25 μg/mL), ENNA (0.5 μg/mL), ENNA_1_ (0.5 μg/mL), BEA (0.5 μg/mL), TEN (0.5 μg/mL), AME (0.5 μg/mL), OT-alpha (1.25 μg/mL), AOH (2.5 μg/mL), ALT (5 μg/mL), and TeA (5 μg/mL). Both solutions were stored at –30 °C.

Methanol (MeOH, LC-MS grade), ACN (LC-MS grade), formic acid (Fac, HPLC grade), aqueous solution of ammonium hydroxide (25%, HPLC grade), and ammonium formate (HPLC grade) were obtained from Fisher Scientific (Leicestershire, UK). Water (LC-MS grade) was purchased from Merck (Darmstadt, Germany). Beta-glucuronidase (Type IX from *E. coli*) was purchased from Sigma-Aldrich (St. Louis, MO, USA). Monopotassium phosphate and dipotassium phosphate were obtained from Acros Organics (Geel, Belgium). All other chemicals and reagents used were of analytical grade or better. An enzyme solution containing 2000 U/mL β-glucuronidase was prepared fresh in phosphate buffer (75 mM, pH 6.8). The Oasis HLB SPE columns (6cc, 200 mg) were purchased from Waters (Milford, MA, USA).

### 4.2. Samples

Mid-stream urine samples were collected in the morning from 60 healthy volunteers (4–70 years, 26 males and 34 females) in Beijing province, immediately transferred to the laboratory and stored at −80 °C. The ethics committee of China National Center for Food Safety Risk Assessment approved the study (No. 2018007, 15 Mar 2018), and the methods were performed according to the approved guidelines and regulations. All the participants were completely informed of the intent of this research. Informed written consents were obtained from the adult participants or parents on behalf of their children prior to inclusion in the study.

### 4.3. Preparation of Standard Soultions and Quality Control Samples

Serial calibration standard solutions at levels ranged from 0.001 to 500 ng/mL for different analytes (Table 3) were prepared in ACN/0.2%FAc (50/50, v/v) by diluting the mixed standard solution in series. Each calibration standard solution contained 2 ng/mL ^13^C-OTA, 2 ng/mL ^13^C-FB_1_, 1 ng/mL ^13^C-FB_2_, 1 ng/mL ^13^C-FB_3_, 30 ng/mLTeA-d_13_, 10 ng/mL AOH-d_2_, 1 ng/mL AME-d_3_, and 2 ng/mL TEN-d_3_. They were prepared fresh as needed. Quality control (QC) samples were obtained by spiking analyte-free urine with the above standard mixtures to reach three (low, medium, and high) concentrations the same as in Table 4. QC samples were measured in each batch of test samples, and their measured values should be within ±15% of the theoretical measurement values.

### 4.4. Sample Preparation

Prior to the extraction of mycotoxins in urine, urine samples were thawed at room temperature and centrifuged at 6000 rpm for 15 min at 4 °C. Fifty microliters of each mixed IS solution were added to 5 mL of the supernatant. The supernatant was incubated with 5 mL phosphate buffer (75 mM, pH 6.8) containing β-glucuronidase (2000 U/mL) in a water bath with gentle shaking for 16 h at 37 °C to allow digestion of mycotoxin conjugates. The mixture was cooled to room temperature and centrifuged again (9500 rpm, 10 min, 4 °C). The pH of the supernatant was adjusted to approximately 3 with formic acid and vortexed for 30 s. The processed sample solutions were loaded onto Oasis HLB SPE columns for further purification. The columns were equilibrated with 5 mL methanol, 5 mL acetonitrile and 5 mL water in turn before sample loading. The column was washed with 5 mL of methanol/water (20/80, v/v), and analytes were eluted sequentially with 5 mL methanol and then 5 mL acetonitrile. The entire 10 mL eluent was dried under nitrogen at 40 °C, reconstituted in 1 mL acetonitrile/water containing 0.2% FAc (50/50, v/v), vortexed for 60 s and centrifuged (4 °C, 20,000 rpm, 30 min). Finally, 200 μL of the supernatant was transferred to vials for LC-MS/MS analysis. The sample preparation procedure resulted in a 5-fold enrichment of the analytes. To determine free forms of mycotoxins, 5 mL of the supernatant from urine sample was diluted with phosphate buffer (75 mM, pH 6.8) of equal volume. This mixture was not combined with β-glucuronidase. The remaining pre-treatment steps are the same as above for total mycotoxins.

### 4.5. LC-MS/MS Conditions

Instrumental method development and practical application for sample analysis were carried out using an Exion LC AD™ System (AB SCIEX, Concord, ON, Canada), coupled with a Sciex Triple Quad ^®^6500 + LC-MS/MS system equipped with a Turbo V electrospray ionization (ESI) source. The Analyst (version 1.6.3) and MultiQuant^®^ 3.0.2 software programs (AB SCIEX, Concord, ON, Canada) were used for instrument control and data evaluation, respectively.

#### 4.5.1. Chromatographic Conditions

Using a gradient elution, analytes of interest were separated on a UHPLC column (CORTECS™ C18, 2.1 × 100 mm, 1.6 μm, Waters, Milford, MA, USA). In order to obtain higher sensitivity for each compound, all the compounds analyzed were divided into two groups with different pH values. The first group consisted of FB_1,_ FB_2_, FB_3_, HFB_1_, HFB_2_, OTA, and OTB, and the mobile phase consisted of solvent A (water acidified with 0.2% FAc) and solvent B (MeOH/ACN, 50/50, v/v). After an incipient period of 1.0 min at 10% B, B increased to 90% within 1–4 min, followed by a hold time of 1.9 min. Finally, eluent B reduced to 10% within 0.1 min, and the column was re-equilibrated at 90% A for 2 min. The total run time was 9 min. For the second group of compounds, acetonitrile and aqueous solution containing 5 mmol/L ammonia acetate and 0.01% ammonium hydroxide were used as mobile phases A and B, respectively. The gradient program was as follows: 10% B at 0–1.0 min, 10%–100% B at 1.0–5.5 min, 100% B at 5.5–7.5 min, and 10% B at 7.6–10 min. The column temperature was 40 °C, and the flow rate was 0.4 mL/min. The injection volume was 5 μL.

#### 4.5.2. Mass Spectrometry Condition

Both positive and negative ionization modes were run simultaneously by quickly switching polarity during the analysis. ESI-MS/MS analysis in scheduled multiple reaction-monitoring (sMRM) mode allowed for quantification of these compounds, using internal standards. Analyte-related MS/MS parameters were optimized via directly injection of individual standard via a peristaltic pump. Parameters of all conducted measurements are displayed in Table 1 and Table 2.

### 4.6. Method Validation

The established method (involving enzyme hydrolysis, SPE purification, and UHPLC-MS/MS analysis) was validated to assess its performance according to Commission Decision 2002/657/EC [46] and the guidelines of European Medicines Agency (EMEA) [47] and Food and Drug Administration (FDA) [48]. The validation was conducted for each of the 18 target mycotoxins, and the main parameters evaluated included linearity, method recovery, precision (intra and inter-day variability), LOD and LOQ.

The linearity was assessed in the range from the LOQ up to 25–500 ng/mL for different analytes by analyzing calibration standards at ten concentrations on three consecutive days, using linear regression with 1/x weighting. LOD and LOQ of each analyte in urine was evaluated by spiking blank urine matrix at low concentration levels that generated signal-to-noise ratios (S/N) of 3 and 10, respectively. Method recovery (R_M_) and precision were investigated at low (0.05–1 ng/mL for different analytes), medium (0.5–10 ng/mL), and high (2–40 ng/mL) spiking levels in blank urine in six replicates with internal standards correction. Evaluation of inter-day precision was performed on three different days. Relative standard deviation (RSD) was calculated to represent precision.

Matrix effects (SSE), extraction recovery (R_E_), and apparent recovery (R_A_) were evaluated by three sets of calibration curves without internal standard correction [49]: Matrix-matched calibration curves prepared by spiking mycotoxin standard solutions into blank urine before (A) and after (B) sample preparation and a calibration curve prepared in ACN/0.2%FAc (50/50) (C). Each calibration curve consisted of five concentration levels in three replicates each. Curve B and C were directly injected for UHPLC-MS/MS analysis. Curve A was subjected to the whole sample preparation procedure prior to instrument analysis, which lead to a 5-fold enrichment of analytes. Therefore, the spiking levels of curve B and C (ranged 0.05–200 ng/mL) were 5 times higher than that of curve A (ranged 0.01–40 ng/mL) to achieve the same theoretical concentration in measurement solutions. The values of SSE, R_A_, and R_E_ for the analytical methods were calculated using the following formulas:

SSE (%) = (slope of calibration curve B)/(slope of calibration curve C),(1)

R_E_ (%) = (slope of calibration curve A)/(slope of calibration curve B), (2)

R_A_ (%) = (slope of calibration curve A)/(slope of standard curve C). (3)

## Figures and Tables

**Table 1:** MRM transitions and MS/MS parameters for 18 mycotoxin analytes.

Compound	Precursor Ion	Q1(*m*/*z*)	Q3(*m*/*z*)	CE (eV) ^1^	DP (V) ^2^
OTA	[M + H]^+^	404.1	239.1 ^a^ 358.1	34 21	50 50
OTB	[M + H]^+^	371.1	205.9 ^a^ 188.1	31 35	40 40
OT-alpha	[M − H]^−^	255.0	211.0 ^a^ 167.1	−21 −32	−30 −30
FB_1_	[M + H]^+^	722.3	334.3 ^a^ 352.3	55 51	50 50
FB_2_	[M + H]^+^	706.4	336.1 ^a^ 354.2	50 46	50 50
FB_3_	[M + H]^+^	706.4	336.3 ^a^ 318.1	50 51	50 50
HFB_1_	[M + H]^+^	406.3	334.1 ^a^ 352.1	35 31	40 40
HFB_2_	[M + H]^+^	390.3	336.2 ^a^ 238.1	33 37	35 35
AOH	[M − H]^−^	257.0	215.0 ^a^ 147.0	−34 −43	−130 −130
ALT	[M + H]^+^	291.0	214.0 ^a^ 229.2	−30 −21	−100 −100
TEN	[M + H]^+^	415.3	312.1 ^a^ 256.0	29 40	110 110
TeA	[M − H]^−^	196.1	139.0 ^a^ 112.0	−25 −35	−50 −50
AME	[M − H]^−^	271.0	256.0 ^a^ 228.0	−30 −36	−110 −110
BEA	[M + H]^+^	784.5	244.2 ^a^ 134.3	41 92	240 240
ENNA	[M + H]^+^	682.4	210.2 ^a^ 228.2	34 38	220 220
ENNA_1_	[M + H]^+^	668.3	210.0 ^a^ 228.1	32 33	200 200
ENNB	[M + H]^+^	640.3	196.4 ^a^ 241.2	34 33	180 180
ENNB_1_	[M + H]^+^	654.3	196.0 ^a^ 210.2	33 35	180 180
^13^C_20_-OTA	[M + H]^+^	424.2	250.1 ^a^ 337.1	35 20	50 50
^13^C_34_-FB_1_	[M + H]^+^	756.7	374.4 ^a^ 356.3	53 55	50 50
^13^C_34_-FB_2_	[M + H]^+^	740.7	358.3 ^a^ 376.2	50 47	50 50
^13^C_34_-FB_3_	[M + H]^+^	740.7	358.2 ^a^ 376.1	50 47	50 50
TeA-d_13_	[M − H]^−^	210.3	143.0 ^a^ 115.0	−27 −34	−50 −50
TEN-d_3_	[M + H]^+^	418.2	314.9 ^a^ 305.4	30 19	140 140
AME-d_3_	[M − H]^−^	274.0	256.0 ^a^ 228.1	−32 −39	−110 −110
AOH-d_2_	[M + H]^+^	258.9	215.1 ^a^ 148.0	−31 −43	−130 −130

^a^ Quantification ion; ^1^ CE, collision energy (eV); ^2^ DP, declustering potential (V).

**Table 2:** Ion source parameters for mycotoxin biomarker analysis.

Ion Source Parameters	Settings
Curtain gas CUR (psi)	20
Collision Gas CAD	10
Ion Spray Voltage (IS) (V)	5500/−4500
Temperature (TEM) (°C)	600
Ion Source Gas 1 (GS1) (psi)	65
Ion Source Gas 2 (GS2) (psi)	55

**Table 3:** Sensitivity, extraction recovery, matrix effect and linearity of each analyte.

Analyte	R_E_ (%)	SSE (%)	R_A_ (%)	Linear Range ^1^ (ng/mL)	R^2^	LOD ^2^ (ng/mL)	LOQ ^3^ (ng/mL)
OTA	107%	49.1%	52.3%	0.05–50	0.999	0.01	0.02
OTB	91.6%	64.3%	58.9%	0.25–250	0.998	0.01	0.04
FB_1_	93.7%	77.3%	72.4%	0.25–250	0.998	0.02	0.05
FB_2_	93.7%	69.3%	64.9%	0.25–250	0.999	0.02	0.05
FB_3_	93.5%	95.7%	89.5%	0.25–250	0.998	0.01	0.03
HFB_1_	104%	64.2%	66.6%	0.25–125	0.998	0.01	0.02
HFB_2_	111%	56.9%	63.0%	0.25–250	0.997	0.02	0.04
TeA	87.3%	41.8%	36.4%	0.5–500	0.999	0.2	0.5
OT-alpha	99.0%	12.2%	12.1%	0.25–125	0.998	0.02	0.05
AOH	75.2%	16.4%	12.4%	0.25–250	0.998	0.01	0.04
ALT	85.2%	93.4%	79.6%	0.5–500	0.998	0.02	0.07
AME	74.6%	120%	89.3%	0.01–50	0.999	0.001	0.003
TEN	88.4%	49.7%	43.9%	0.05–50	0.998	0.002	0.01
ENNB	90.0%	101%	90.8%	0.001–25	0.998	0.0001	0.0002
ENNB_1_	84.1%	104%	87.1%	0.001–25	0.997	0.0001	0.0002
ENNA	71.6%	101%	72.5%	0.005–50	0.997	0.0002	0.0008
ENNA_1_	77.5%	101%	78.5%	0.005–50	0.998	0.0001	0.0005
BEA	85.5%	89.7%	76.6%	0.005–50	0.998	0.0002	0.0006

^1^ Linear range of calibration curves prepared in pure solvent. ^2^ Limit of detection in urine. ^3^ Limit of quantification in urine.

**Table 4:** Recovery and precision of the developed method in spiked urine samples (*n* = 6).

Analyte	Spiked Level in Urine (ng/mL)	Measured Value in Urine (ng/mL)	Method Recovery (%)	RSD (%)	
				Intra-day (*n* = 6)	Inter-day (*n* = 18)
OTA	0.1	0.10	100	2.8	4.8
	1	1.11	111	3.3	3.5
	4	4.15	104	2.4	3.8
OTB	0.5	0.41	82.1	1.8	10.8
	5	4.18	83.5	2.8	4.6
	20	17.4	86.9	0.9	5.8
FB_1_	0.5	0.47	94.6	3.1	3.8
	5	4.95	99.0	3.6	4.9
	20	19.5	97.4	2.5	3.4
FB_2_	0.5	0.49	98.0	3.7	4.8
	5	5.28	106	3.8	10.0
	20	18.3	91.7	3.5	6.5
FB_3_	0.5	0.47	93.3	2.7	4.3
	5	5.20	104	4.6	6.3
	20	19.4	97.1	2.5	6.0
HFB_1_	0.25	0.21	83.5	0.9	3.6
	2.5	2.25	89.8	0.5	5.1
	10	8.83	88.3	3.5	5.6
HFB_2_	0.5	0.37	74.0	3.1	9.5
	5	4.22	84.5	1.1	3.5
	20	16.4	82.0	0.6	6.6
TeA	1	0.93	93.0	6.9	9.2
	10	9.84	98.4	6.2	8.3
	40	38.3	95.8	1.3	4.4
OT-alpha	0.25	0.21	84.4	1.2	8.7
	2.5	2.00	79.9	4.1	6.5
	10	0.88	88.1	1.9	8.7
AOH	0.5	0.538	108	1.4	7.5
	5	5.33	107	2.2	4.4
	20	21.3	107	2.2	2.7
ALT	1	1.15	115	8.7	13.4
	10	13.3	133	6.3	11.1
	40	51.8	130	3.2	7.3
AME	0.1	0.08	80.9	2.7	5.2
	1	0.92	92.2	0.7	2.6
	4	3.81	95.2	1.7	2.4
TEN	0.1	0.11	110	4.8	6.0
	1	1.08	108	2.7	2.8
	4	4.12	103	1.2	3.2
ENNB	0.05	0.05	94.6	1.7	4.9
	0.5	0.49	98.5	2.8	4.9
	2	2.02	101	2.7	4.3
ENNB_1_	0.05	0.05	95.0	6.9	7.2
	0.5	0.49	97.4	1.1	5.9
	2	1.92	96.0	3.2	6.2
ENNA	0.1	0.09	84.3	1.7	8.2
	1	0.90	89.9	3.6	7.0
	4	3.79	94.7	3.2	6.9
ENNA_1_	0.1	0.09	90.0	2.9	9.9
	1	1.00	99.4	1.4	7.5
	4	3.90	97.5	3.8	7.5
BEA	0.1	0.08	80.8	4.1	5.8
	1	0.77	76.4	1.8	3.9
	4	3.20	80.1	2.7	4.3

**Table 5:** Analytical methods for the determination of mycotoxins in urine.

Analyte	Sample Preparation	Analysis Method	LOQ (μg/L)	Reference
OTA, OT-alpha, FB_1_, FB_2_, FB_3_ HFB_1_, AOH, AME	QuEChERS	UPLC-MS/MS	0.02–1.0	[15]
OTA, OT-alpha, FB_1_, ENNB	Dilute and shoot	LC-MS/MS	0.0005–0.1	[43]
OTA, FB_1_, AOH	SPE	UHPLC-MS/MS	0.001–0.03	[3]
OTA, FB_1_	LLE+ QuEChERS	UPLC–MS/MS	0.16–1.1	[36]
OTA, OT-alpha, FB_1_	Direct method	UHPLC-MS/MS	0.1–0.5	[8]
OTA, OT-alpha	LLE	HPLC-FD	0.02	[32]
OTA, OT-alpha, FB_1_, FB_2_, FB_3_	Direct method/ICA	LC–MS/MS	0.003–0.2	[41]
OTA, FB_1_, FB_2_	Dilute and shoot	LC–MS/MS	0.017–0.17	[4]
OTA, FB_1_, FB_2_	SPE + SPE	LC–MS/MS	0.007–0.017	[19]
ENNB	salting-out liquid–liquid extraction	UHPLC-Q-Orbitrap HRMS	0.001	[38]
ENN_S_, BEA	SPE	LC-MS/MS	0.005–0.02	[7]
OTA, OT-alpha, FB_1_, HFB_1_	LLE + SPE	LC-MS/MS	0.06–1.02	[14]

**Table 6:** Occurrence of mycotoxin biomarkers in urine samples with enzyme treatment (*n* = 60).

Mycotoxin Biomarkers	Total Concentrations			
	Positive (%)	Mean ± SD (ng/mL)	Range (ng/mL)	Median (ng/mL)
OTA	24 (40%)	0.02 ± 0.02	<LOD~0.14	0.005
OT-alpha	20 (33.3%)	0.22 ± 0.42	<LOD~2.38	0.001
FB_1_	2 (3.3%)	0.01 ± 0.01	<LOD~0.07	0.01
FB_3_	1 (1.7%)	0.01	<LOD~0.01	0.005
HFB_1_	1 (1.7%)	0.01 ± 0.01	<LOD~0.09	0.005
TeA	52 (86.7%)	4.4 ± 8.7	<LOD~54.1	1.4
AOH	17 (28.3%)	0.34 ± 1.09	<LOD~7.68	0.01
AME	50 (83.3%)	0.059 ± 0.049	<LOD~0.167	0.048
TEN	23 (38.3%)	0.024 ± 0.042	<LOD~0.193	0.001
ENNB	24 (40.0%)	0.0002 ± 0.0002	<LOD~0.0014	0.0001

For calculation of mean, standard deviation and median values, concentration <LOD was assigned half the LOD.
